# Supplementary material for: Intranasal influenza-vectored COVID-19 vaccine restrains the SARS-CoV-2 inflammatory response in hamsters
Source: Nat Commun. 2023 Jul 11;14:4117. doi: 10.1038/s41467-023-39560-9 (PMC10336035; doi:10.1038/s41467-023-39560-9)
Supplement: Supplementary file 1 — Supplementary Information [file 41467_2023_39560_MOESM1_ESM.pdf]

Supplementary Materials

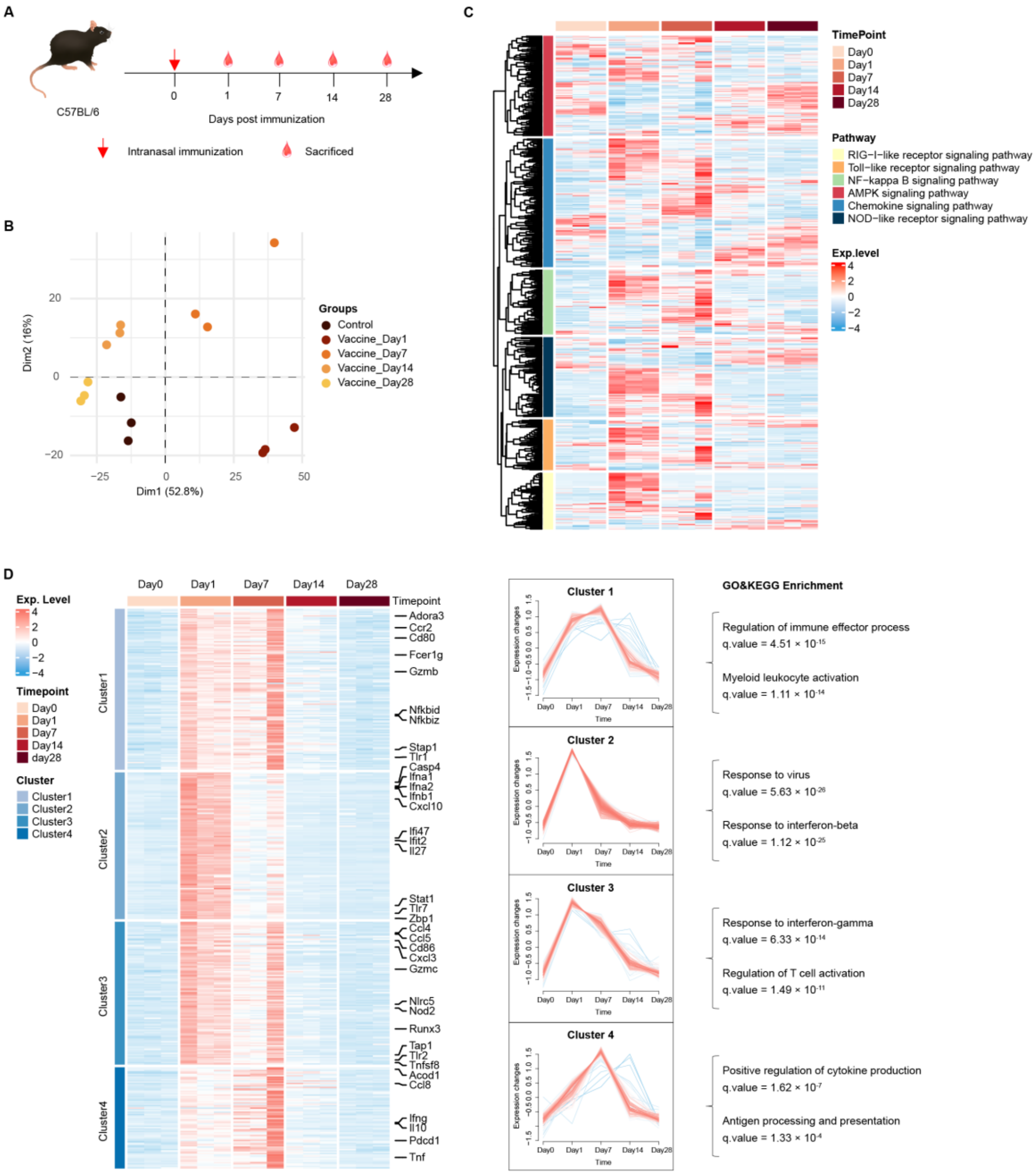

Fig S1. Transcriptional dynamics show activated antiviral innate immune responses in the lung induced by dNS1-RBD.

(A) Experimental Schema, created with adobe illustrator 2020.

(B) Principal component analysis (PCA) of the data collected from the lung of C57BL/6 mice at 1-, 7-, 14-, and 28- days post-vaccination and control mice (dark brown). n = 3 biologically independent mice/group.

(C). Heatmap visualization of scaled gene expression levels (TPM) for selected pathways of interest.

(D) Heatmap showing the dynamic expression patterns of DEGs. Expression trends of the genes in the four clusters generated by time series clustering and related GO and KEGG enrichment results are shown in the right part.

GO: Gene Ontology; KEGG: Kyoto Encyclopedia of Genes and Genomes.

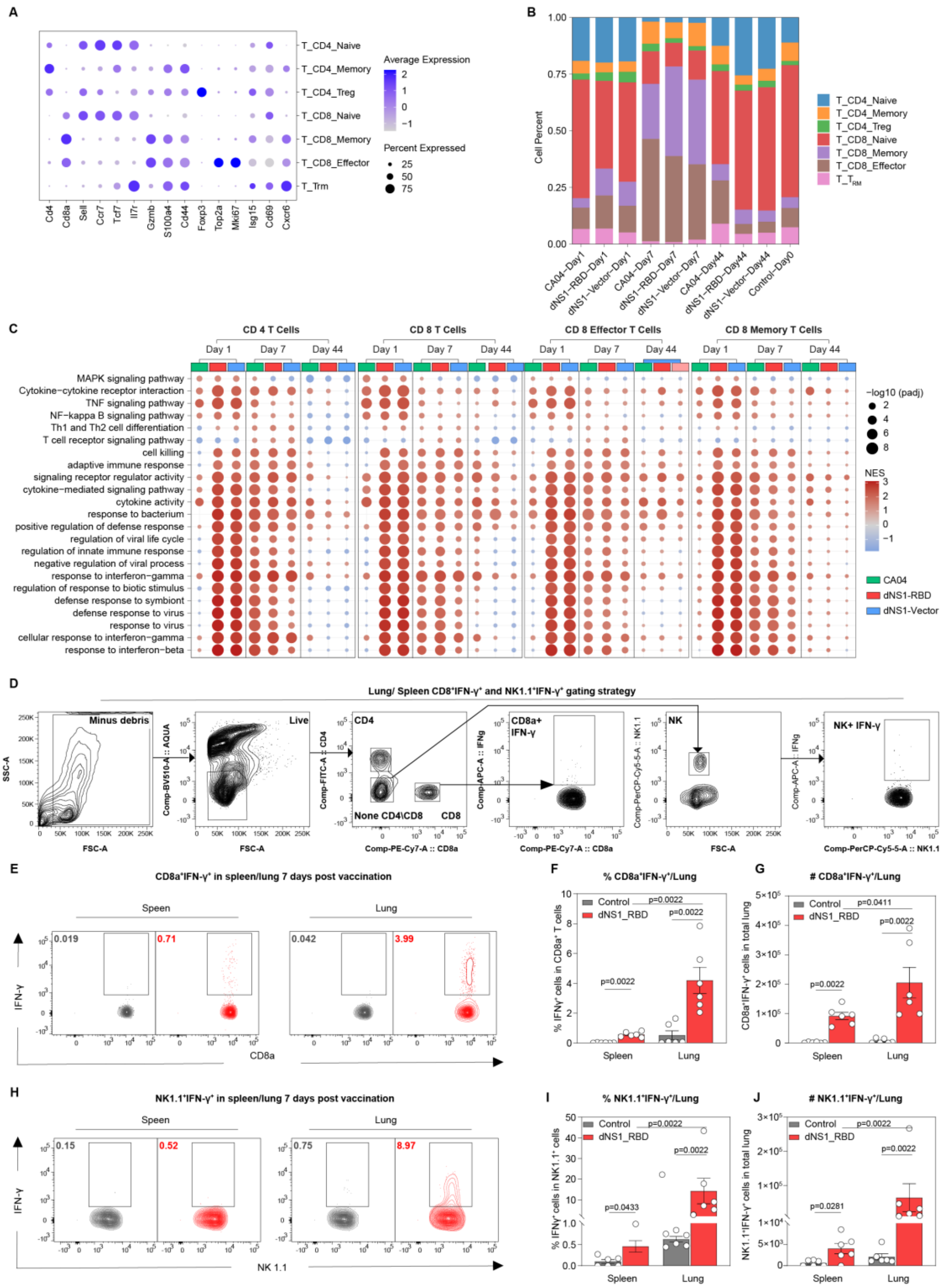
A
T_CD4_Naive
T_CD4_Memory
T_CD4_Treg
T_CD8_Naive
T_CD8_Memory
T_CD8_Effector
T_Trm
Average Expression
Percent Expressed
Cd4 Cd8a Sell Ccr7 Tcf7 Il7r Gzmb S100a4 Cd44 Foxp3 Top2a Mki67 Isg15 Cd69 Cxcr6
B
Cell Percent
CA04-Day1 dNS1-RBD-Day1 dNS1-Vector-Day1 CA04-Day7 dNS1-RBD-Day7 dNS1-Vector-Day7 CA04-Day44 dNS1-RBD-Day44 dNS1-Vector-Day44 Control-Day0
C
CD 4 T Cells
CD 8 T Cells
CD 8 Effector T Cells
CD 8 Memory T Cells
Day 1
Day 7
Day 44
MAPK signaling pathway
Cytokine−cytokine receptor interaction
TNF signaling pathway
NF−kappa B signaling pathway
Th1 and Th2 cell differentiation
T cell receptor signaling pathway
cell killing
adaptive immune response
signaling receptor regulator activity
cytokine−mediated signaling pathway
cytokine activity
response to bacterium
positive regulation of defense response
regulation of viral life cycle
regulation of innate immune response
negative regulation of viral process
response to interferon−gamma
regulation of response to biotic stimulus
defense response to symbiont
defense response to virus
response to virus
cellular response to interferon−gamma
response to interferon−beta
−log10 (padj)
NES
CA04
dNS1-RBD
dNS1-Vector
D
Lung/ Spleen CD8+IFN-γ+ and NK1.1+IFN-γ+ gating strategy
Minus debris
Live
CD4
None CD4\CD8
CD8
CD8a+ IFN-γ
NK
NK+ IFN-γ
SSC-A
FSC-A
Comp-BV510-A :: AQUA
Comp-FITC-A :: CD4
Comp-PE-Cy7-A :: CD8a
Comp-APC-A :: IFNg
Comp-PerCP-Cy5-5-A :: NK1.1
E
CD8a+IFN-γ+ in spleen/lung 7 days post vaccination
Speen
Lung
0.019
0.71
0.042
3.99
IFN-γ
CD8a
F
% CD8a+IFN-γ+/Lung
Control
dNS1_RBD
% IFNγ+ cells in CD8a+ T cells
p=0.0022
Spleen
Lung
G
CD8a+IFN-γ+/Lung
CD8a+IFN-γ+ cells in total lung
p=0.0411
p=0.0022
H
NK1.1+IFN-γ+ in spleen/lung 7 days post vaccination
0.15
0.52
0.75
8.97
NK 1.1
I
% NK1.1+IFN-γ+/Lung
% IFNγ+ cells in NK1.1+ cells
p=0.0433
J
NK1.1+IFN-γ+/Lung
NK1.1+IFN-γ+ cells in total lung
p=0.0281

Fig. S2 dNS1-RBD induces SARS-CoV2-specific T-cell response and reveals a robust transcriptional response.

(A) Dot plots of cells expressing selected canonical marker genes for identification of 7 different cell types in T cells across all sample.

(B) Stacked barplot showing the proportion of different T cells across all groups.

(C) Dot plots showing the GO and KEGG gene set enrichment analysis (GSEA) results of CD4 T cell, CD8 T cells, CD8 Effector T cell and CD8 Memory T cell.

(D) Gating strategy for NK cells and IFN-γ^+ $CD8^+$ T cells in the lung and spleen, remove doublets and $CD45^+$ cell gating are not shown

(E) Representative flow cytometry contour plots showing IFN-γ produced by $CD8^+$ T cells in the lung and spleen at 7 days post-immunization. Control (gray), dNS1-RBD (red).

(F-G) Bar graph showing frequency (F) and absolute number (G) of IFN-γ-producing $CD8^+$ T cells in the lung and spleen. n = 6 biologically independent mice/group. Control (gray), dNS1-RBD (red).

(H) Flow cytometry analysis of IFN-γ-producing $NK1.1^+$-cells in the lung and spleen at 7 days post-immunization. Control (gray), dNS1-RBD (red).

(I-J) Bar graph depicting frequency (I) and absolute number (J) of $NK1.1^+$ IFN-γ^+ cells in the lung and spleen at 7 days post-immunization. n = 6 biologically independent mice/group. Control (gray), dNS1-RBD (red).

Data are presented as mean ± SEM. Statistics analysis were Mann-Whitney tests (two-sided). Source data are provided as a Source Data file. GO: Gene Ontology; KEGG: Kyoto Encyclopedia of Genes and Genomes; ssGSEA: single cell gene-set enrichment analysis.

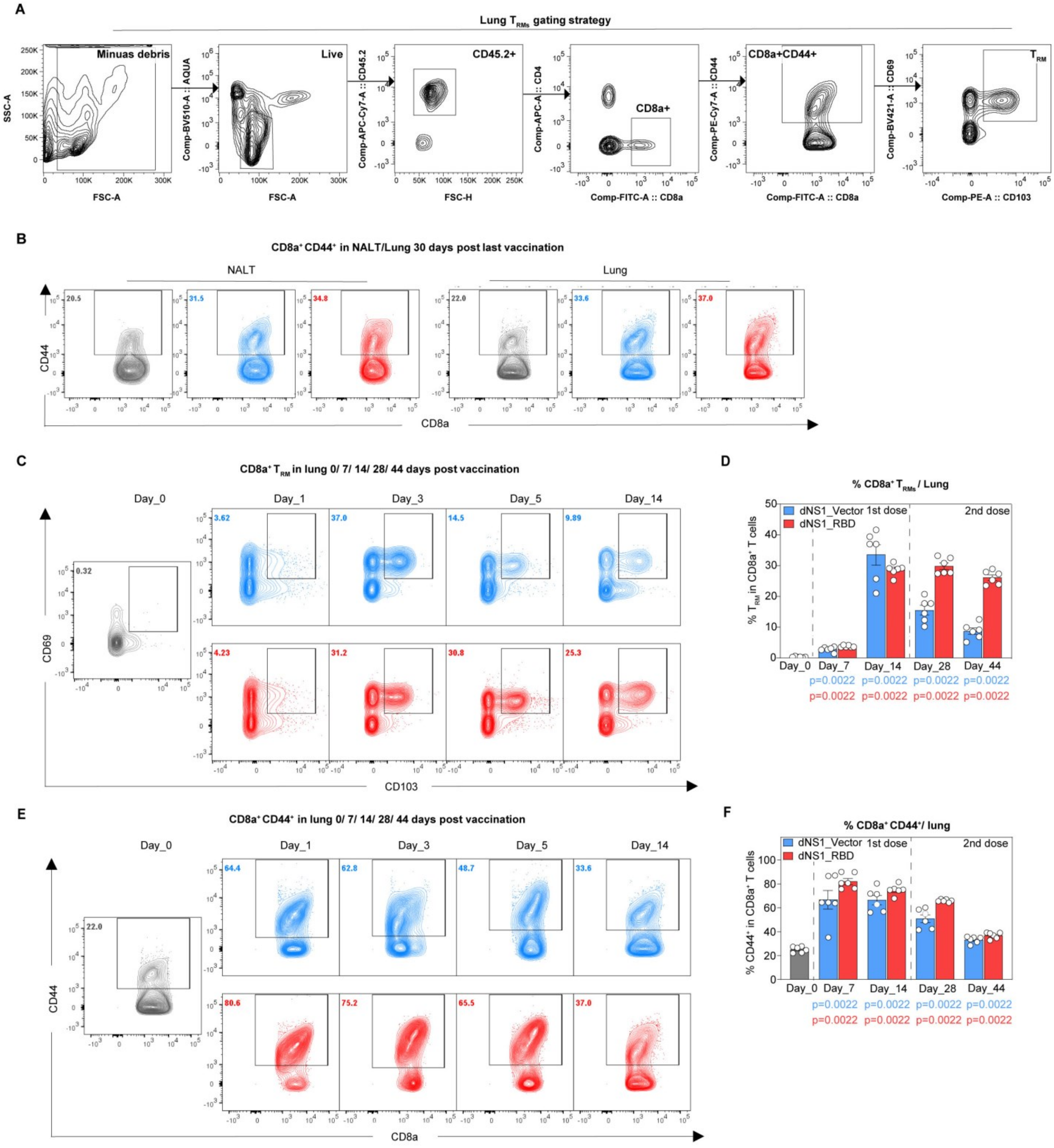

A
Lung T_{RMs} gating strategy
Minuas debris
Live
CD45.2+
CD8a+
CD8a+CD44+
T_{RM}
SSC-A
FSC-A
Comp-BV510-A :: AQUA
Comp-APC-Cy7-A :: CD45.2
FSC-H
Comp-APC-A :: CD4
Comp-FITC-A :: CD8a
Comp-PE-Cy7-A :: CD44
Comp-BV421-A :: CD69
Comp-PE-A :: CD103
B
$CD8a^+ CD44^+$ in NALT/Lung 30 days post last vaccination
NALT
Lung
20.5
31.5
34.8
22.0
33.6
37.0
CD44
CD8a
C
$CD8a^+ T_{RM}$ in lung 0/ 7/ 14/ 28/ 44 days post vaccination
Day_0
Day_1
Day_3
Day_5
Day_14
0.32
3.62
37.0
14.5
9.89
4.23
31.2
30.8
25.3
CD69
CD103
D
% $CD8a^+ T_{RMs}$ / Lung
dNS1_Vector
dNS1_RBD
1st dose
2nd dose
% T_{RM} in $CD8a^+$ T cells
Day_0
Day_7
Day_14
Day_28
Day_44
p=0.0022 p=0.0022 p=0.0022 p=0.0022
p=0.0022 p=0.0022 p=0.0022 p=0.0022
E
$CD8a^+CD44^+$ in lung 0/ 7/ 14/ 28/ 44 days post vaccination
Day_0
Day_1
Day_3
Day_5
Day_14
22.0
64.4
62.8
48.7
33.6
80.6
75.2
65.5
37.0
CD44
CD8a
F
% $CD8a^+ CD44^+$/ lung
dNS1_Vector
dNS1_RBD
1st dose
2nd dose
% $CD44^+$ in $CD8a^+$ T cells
Day_0
Day_7
Day_14
Day_28
Day_44
p=0.0022 p=0.0022 p=0.0022 p=0.0022
p=0.0022 p=0.0022 p=0.0022 p=0.0022

Fig. S3 dNS1-RBD induces tissue-resident memory T cells cover upper and lower respiratory tract in mice.

(A) Gating strategy for $CD44^+$ $CD8a^+$ T cells and T_{RM} in the lung and NALT, remove doublets is not shown.

(B) Representative flow cytometry contour plots for $CD44^+$ $CD8a^+$ T cells in NALT and lung. Control (gray), dNS1-Vector (blue), dNS1-RBD (red).(C) Representative flow cytometry contour plots for T_{RM} in the lung. Control (gray), dNS1-Vector (blue), dNS1-RBD (red).

(D) Bar graph showing frequency of T_{RM} in the lung at indicated time points. n = 6 biologically independent mice/group. Control (gray), dNS1-Vector (blue), dNS1-RBD (red).

(E) Representative flow cytometry contour plots for $CD44^+$ $CD8a^+$ T cells in the lung. Control (gray), dNS1-Vector (blue), dNS1-RBD (red).

(F) Bar graph showing frequency of $CD44^+$ $CD8a^+$ T cells in the lung at indicated time points. n = 6 biologically independent mice/group. Control (gray), dNS1-Vector (blue), dNS1-RBD (red).

Data are presented as mean ± SEM. Statistics analysis were Mann-Whitney tests (two-sided). Source data are provided as a Source Data file. T_{RM}: tissue-resident memory T cells; NALT: Nasal-associated lymphoid tissue.

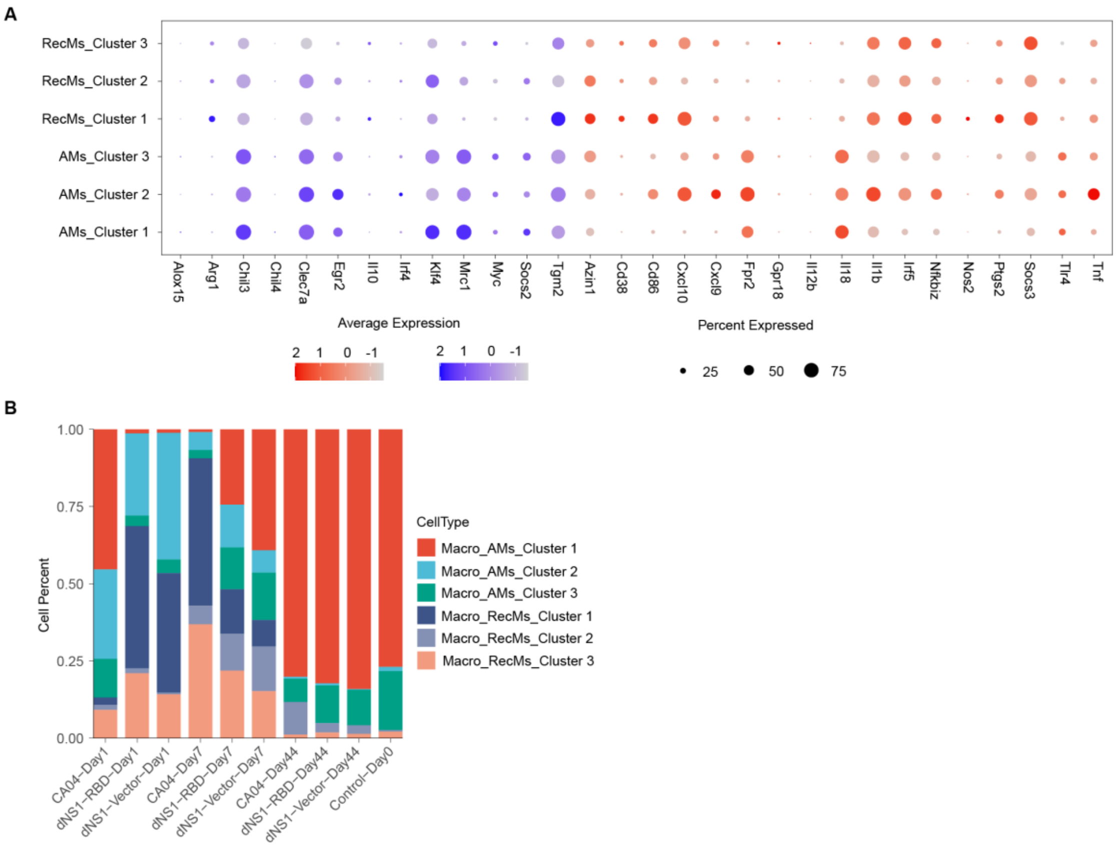

Fig. S4 dNS1-RBD reveals a robust transcriptional response in macrophages and monocytes.

(A) Dot plots of M1 (red) or M2 (blue) marker genes in macrophages.

(B) Stacked barplot showing the proportion of different macrophage subtypes.

AMs: alveolar macrophages; RecMs: recruited macrophages.

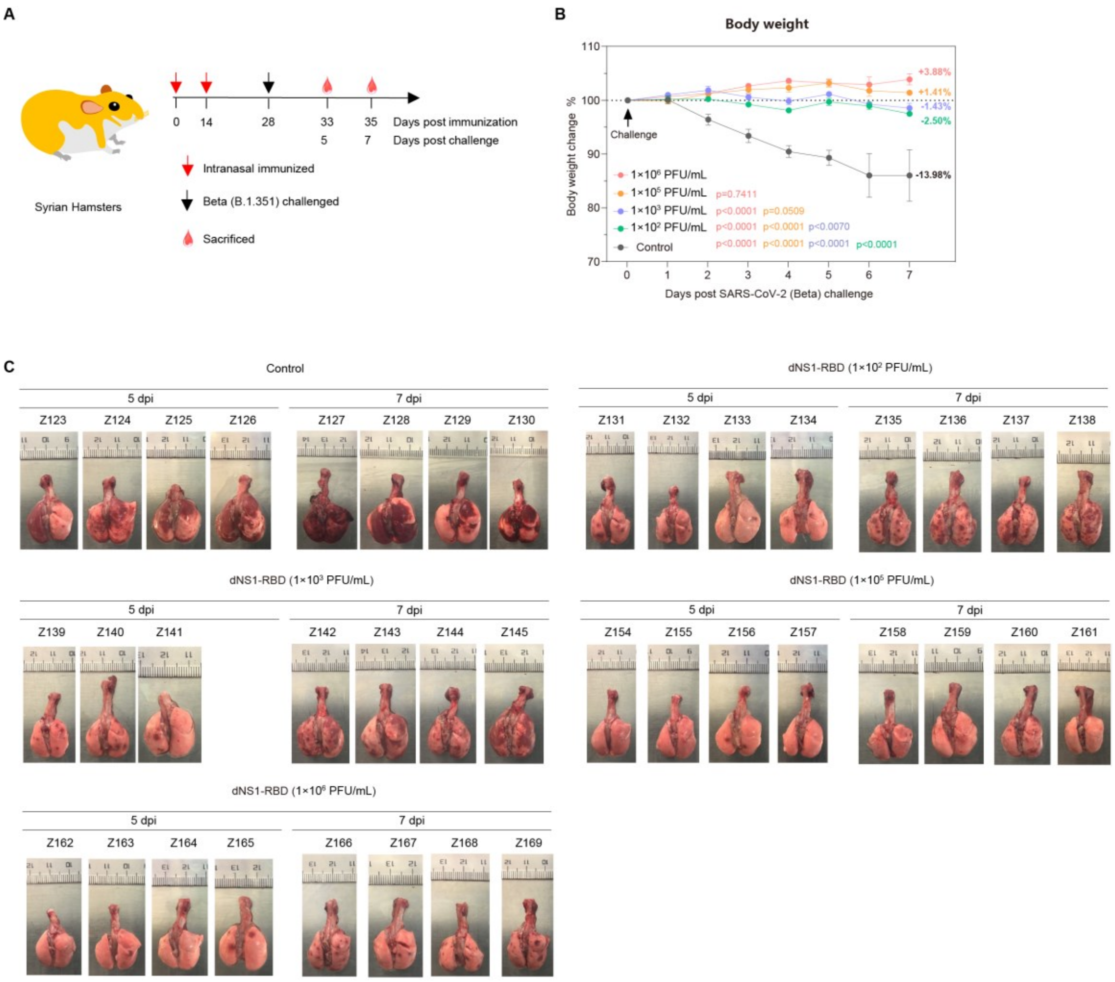

Fig. S5 Hamsters receiving varying doses of the vaccine demonstrated different protective effects against SARS-CoV-2 challenge.

(A) Schema of the experimental design, created with adobe illustrator 2020.

(B) Body weight changes of hamsters after cohoused exposure were plotted. The average weight loss of each group at 7 dpi is indicated as a colored number, n = 7-8 biologically independent hamsters/group. One hamster in the 1x10^3 PFU/mL group died due to accidental anesthesia before receiving the vaccine vaccination. P-values are shown in the figure using different colors. The 1×10^6 PFU/mL group is represented in pink, the 1×10^5 PFU/mL group in orange, the 1×10^3 PFU/mL group in purple, and the 1×10^2 PFU/mL group in green.

(C) Gross lung images from dNS1-RBD vaccinated (1×10^6 PFU/mL, 1×10^5 PFU/mL, 1×10^4 PFU/mL, 1×10^3 PFU/mL, 1×10^2 PFU/mL) and control groups.

Data are presented as mean ± SEM. Statistical analysis was two-way ANOVA with Bonferroni's multiple comparisons test. ns, non-significant. Source data are provided as a Source Data file.

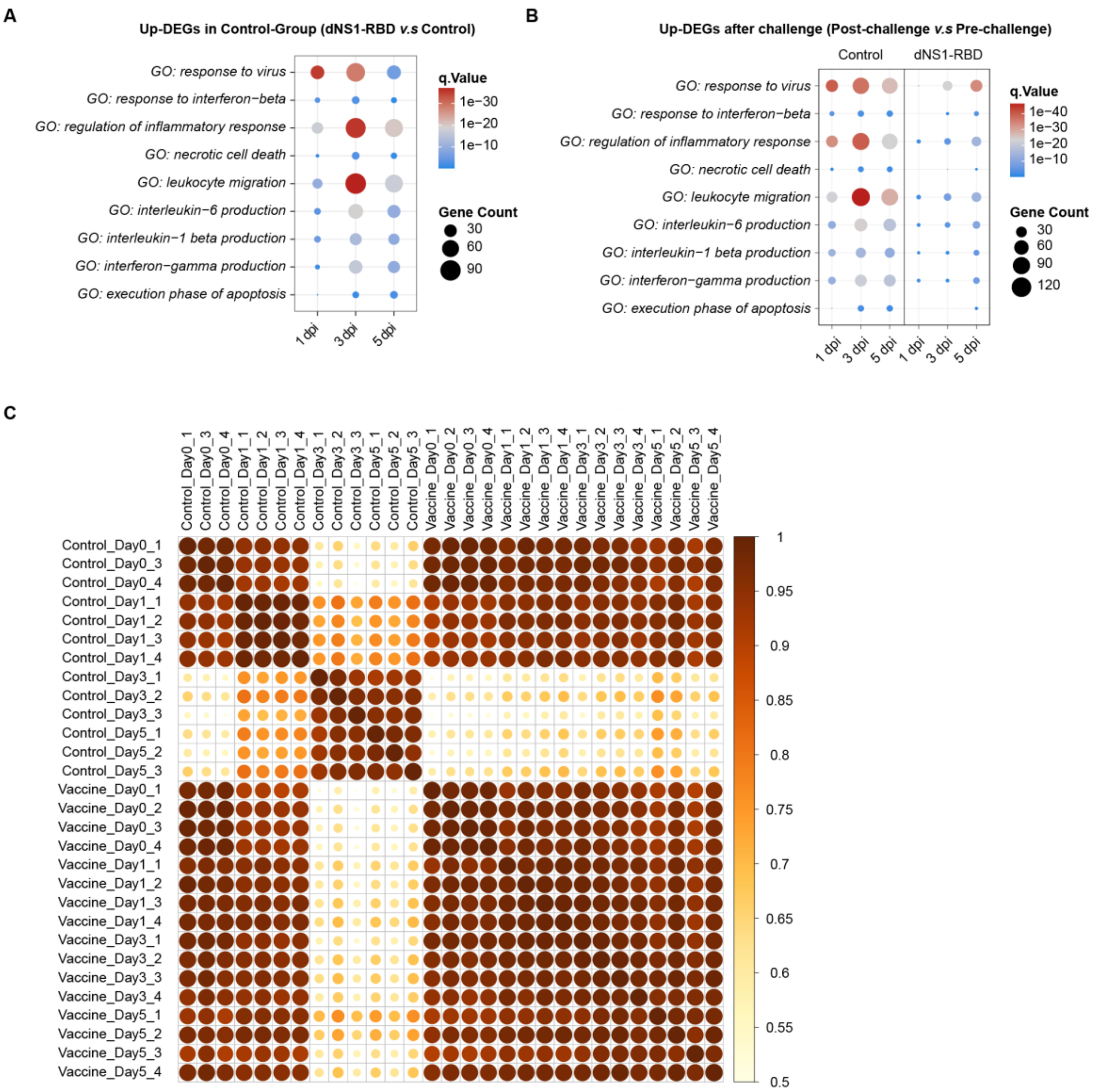

Fig. S6 Intranasal vaccination with dNS1-RBD attenuated the inflammation responses after SARS-Cov-2 challenge.

(A) Dot plots showing the Gene Ontology (GO) enrichment analysis of up-regulated differentially expressed genes (Up-DEGs) related to immune responses in control hamsters compared to vaccinated hamsters.

(B) Dot plots showing the GO enrichment analysis of up-regulated differentially expressed genes (Up-DEGs) related to immune responses compared to 0 dpi in control hamsters and vaccinated hamsters respectively.

(C) Pearson correlation analysis between all biological RNA-sample.
