# Supplementary material for: Intranasal influenza-vectored COVID-19 vaccine restrains the SARS-CoV-2 inflammatory response in hamsters
Source: Nat Commun. 2023 Jul 11;14:4117. doi: 10.1038/s41467-023-39560-9 (PMC10336035; doi:10.1038/s41467-023-39560-9)
Supplement: Supplementary file 3 — Reporting Summary [file 41467_2023_39560_MOESM3_ESM.pdf]

nature portfolio

Corresponding author(s): Ningshao Xia

Last updated by author(s): May 27, 2023

Reporting Summary

Nature Portfolio wishes to improve the reproducibility of the work that we publish. This form provides structure for consistency and transparency in reporting. For further information on Nature Portfolio policies, see our Editorial Policies and the Editorial Policy Checklist.

Statistics

For all statistical analyses, confirm that the following items are present in the figure legend, table legend, main text, or Methods section.

n/a	Confirmed	
☐	☒	The exact sample size (*n*) for each experimental group/condition, given as a discrete number and unit of measurement
☐	☒	A statement on whether measurements were taken from distinct samples or whether the same sample was measured repeatedly
☐	☒	The statistical test(s) used AND whether they are one- or two-sided<br>*Only common tests should be described solely by name; describe more complex techniques in the Methods section.*
☒	☐	A description of all covariates tested
☐	☒	A description of any assumptions or corrections, such as tests of normality and adjustment for multiple comparisons
☐	☒	A full description of the statistical parameters including central tendency (e.g. means) or other basic estimates (e.g. regression coefficient) AND variation (e.g. standard deviation) or associated estimates of uncertainty (e.g. confidence intervals)
☐	☒	For null hypothesis testing, the test statistic (e.g. *F*, *t*, *r*) with confidence intervals, effect sizes, degrees of freedom and *P* value noted<br>*Give P values as exact values whenever suitable.*
☒	☐	For Bayesian analysis, information on the choice of priors and Markov chain Monte Carlo settings
☒	☐	For hierarchical and complex designs, identification of the appropriate level for tests and full reporting of outcomes
☐	☒	Estimates of effect sizes (e.g. Cohen's *d*, Pearson's *r*), indicating how they were calculated

Our web collection on statistics for biologists contains articles on many of the points above.

Software and code

Policy information about availability of computer code

Data collection	Flow cytometry data with BD LRFORTESSAX-20 (BD Biosciences) flow cytometry system,and BD FACS Aria Fusion machine for sortingEllSpotdata with CTL S6 Universal Analyzer (Cellular Technology liited)Whole-slide images of the lung sections were captured using a leica AperioVersa 200 microscope.
Data analysis	Flow cytometry data were analyzed using FlowJo X 10.0.7r2Statistical analyses were performed with Prism 8 (GraphPad soft-ware). Barsrepresent the mean. Statistical signifcance of two group wasperformed with Mann-Whitney tests. Statistical sienifcance of three or morethan three was performed with ruska-Wallis tests with Dunn'smultiple comparisons test.And the visualization of RNA-sed and cRNA-seganalysis were performed by using R package ggplot2/version 3.3.6and ComplexHeatmap(version 2.10.0).

For manuscripts utilizing custom algorithms or software that are central to the research but not yet described in published literature, software must be made available to editors and reviewers. We strongly encourage code deposition in a community repository (e.g. GitHub). See the Nature Portfolio guidelines for submitting code & software for further information.

Data

Policy information about availability of data

All manuscripts must include a data availability statement. This statement should provide the following information, where applicable:

- Accession codes, unique identifiers, or web links for publicly available datasets
- A description of any restrictions on data availability
- For clinical datasets or third party data, please ensure that the statement adheres to our policy

The raw and processed data of RNA-seq, ATAC-seq and scRNA-seq experiment generated in this study have been deposited in the Gene Expression Omnibus (GEO) repository under SuperSeries accession code GSE227649 [add hyperlink here:https://www.ncbi.nlm.nih.gov/geo/query/acc.cgi?acc=GSE227649]. Any other raw data or non-commercial material used in this study are available from the corresponding author upon request. Source data are provided with this paper.

Research involving human participants, their data, or biological material

Policy information about studies with human participants or human data. See also policy information about sex, gender (identity/presentation), and sexual orientation and race, ethnicity and racism.

Reporting on sex and gender	The study did not involve human data.
Reporting on race, ethnicity, or other socially relevant groupings	The study did not involve human data.
Population characteristics	The study did not involve human data.
Recruitment	The study did not involve human data.
Ethics oversight	The study did not involve human data.

Note that full information on the approval of the study protocol must also be provided in the manuscript.

Field-specific reporting

Please select the one below that is the best fit for your research. If you are not sure, read the appropriate sections before making your selection.

☒ Life sciences ☐ Behavioural & social sciences ☐ Ecological, evolutionary & environmental sciences

For a reference copy of the document with all sections, see nature.com/documents/nr-reporting-summary-flat.pdf

Life sciences study design

All studies must disclose on these points even when the disclosure is negative.

Sample size	The sample sizes were empirically estimated by considering the variations in the results and the statistical power needed, while minimizing the number of animals used. The sample size was at least 3 or above. For C57BL/6 mice, 6-10 mice were used to determine immune response and 2-3 mice were used to perform RNA-seq, sc-RNA-seq or ATAC-seq. For SARS-CoV-2 challenge experiments, 7-8 hamsters were challenged after being vaccinated and 3-4 hamsters were sacrificed at each time point for tissue viral RNA load and lung pathology and RNAseq.
Data exclusions	No data was excluded.
Replication	All experiments were repeated at least once. For SARS-CoV-2 challenge experiments, they were repeated at least 3 times and similar findings were obtained from all repeats.
Randomization	Animal experiments related to the manuscript were conducted using a completely random grouping method.
Blinding	For all experiments conducted in this study, with the exception of histopathology samples that were analyzed in a blinded manner by a pathologist, the investigators were aware of the group allocation during data collection and analysis. Blinding was not feasible or necessary for other experiments since the results were quantitative and objective, and did not involve subjective judgment.

Reporting for specific materials, systems and methods

We require information from authors about some types of materials, experimental systems and methods used in many studies. Here, indicate whether each material, system or method listed is relevant to your study. If you are not sure if a list item applies to your research, read the appropriate section before selecting a response.

Materials & experimental systems

n/a	Involved in the study
☐	☒ Antibodies
☒	☐ Eukaryotic cell lines
☒	☐ Palaeontology and archaeology
☐	☒ Animals and other organisms
☒	☐ Clinical data
☒	☐ Dual use research of concern
☒	☐ Plants

Methods

n/a	Involved in the study
☒	☐ ChIP-seq
☐	☒ Flow cytometry
☒	☐ MRI-based neuroimaging

Antibodies

Antibodies used

CD4 FITC (GK1.5) Biolegend Cat# 100406; RRID: AB_312691 1:200 for cytometry
CD103 PE (2E7) Biolegend Cat# 121406; RRID: AB_1133989 1:200 for cytometry
CD11b FITC (M1/70) Biolegend Cat# 101206; RRID: AB_312789 1:200 for cytometry
CD11c APC (N418) Biolegend Cat# 117310; RRID: AB_313779 1:200 for cytometry
CD11c BV421 (N418) Biolegend Cat# 117343; RRID: AB_2563099 1:400 for cytometry
CD170 PE (S17007L) Biolegend Cat# 155506; RRID: AB_2750235 1:200 for cytometry
CD4 APC (GK1.5) Biolegend Cat# 100412; RRID: AB_312697 1:200 for cytometry
CD44 PE/Cy7 (IM7) Biolegend Cat# 103030; RRID: AB_830787 1:200 for cytometry
CD45.2 APC/Cy7 (104) Biolegend Cat# 109824; RRID: AB_830789 1:200 for cytometry
CD45.2 PerCp-Cy5.5 (104) Biolegend Cat# 109828; RRID: AB_893350 1:100 for cytometry
CD45.2 FITC (104) Biolegend Cat# 109805; RRID: AB_313442 1:200 for cytometry
CD49d APC (R1-2) Biolegend Cat# 104426; RRID: AB_493719 1:200 for cytometry
CD62L AF700 (MEL-14) Biolegend Cat# 104426; RRID: AB_493719 1:200 for cytometry
CD64 PE/Cy7 (X54-5/7.1) Biolegend Cat# 139314; RRID: AB_2563904 1:200 for cytometry
CD69 BV421 (H1.2F3) Biolegend Cat# 104545; RRID: AB_2686969 1:400 for cytometry
CD8 FITC (53-6.7) Biolegend Cat# 100706; RRID: AB_312745 1:200 for cytometry
CD80 PE/Cy7 (16-10A1) Biolegend Cat# 104734; RRID: AB_2563113 1:200 for cytometry
CD8a PE/Cy7 (53-6.7) Biolegend Cat# 100722; RRID: AB_312761 1:200 for cytometry
CD8b APC/Cy7 (YTS156.7.7) Biolegend Cat# 126619; RRID: AB_2563950 1:200 for cytometry
I-A/I-E APC (M5/114.15.2) Biolegend Cat# 107614; RRID: AB_313329 1:200 for cytometry
IFN-γ APC (XMG1.2) Biolegend Cat# 505810; RRID: AB_315404 1:200 for cytometry
Ly-6C APC/Cy7 (HK1.4) Biolegend Cat# 128026; RRID: AB_10640120 1:200 for cytometry
NK1.1 PerCp-Cy5.5 (PK136) Biolegend Cat# 108728; RRID: AB_2132705 1:100 for cytometry
CD16/CD32 Purified (2.4G2) BD Cat# 553142; RRID: AB_394657 1:100 for cytometry
CD44 PE/Cy7 (1M7) BD Cat# 560569; RRID: AB_1727484 1:200 for cytometry
CD86 BV605 (PO3) BD Cat# 745236; RRID: AB_2742823 1:400 for cytometry

Validation

Antibodies were chosen based on the validation statements for species (mouse) and application (FACS) on themanufacturer'swebsite.

Animals and other research organisms

Policy information about studies involving animals; ARRIVE guidelines recommended for reporting animal research, and Sex and Gender in Research

Laboratory animals

C57BL/6 mice were purchased from Shanghai SLAC Laboratory Animal Co,Ltd.(6-8 week)Golden Syrian hamsters were purchasedfrom Beijing Vital River Laboratory Animal Technology Co., Ltd.(6-8 week);Environmental control: Temperature: 18-29°C, Humidity: 45-55%, Air exchange rate: 6-15 times/hour, Circadian rhythm: 12/12 hours light/dark cycle.

Wild animals

No wild animals were used in this study.

Reporting on sex

Experiments were conducted in hamsters to verify the effectiveness of the vaccine. The experiments were conducted with an equal number of male and female hamsters, which fully demonstrated that the vaccine has well protective effects. Subsequent experiments used only female animals.

Field-collected samples

Field collected samples were not involved in this study.

Ethics oversight

All animal experiments strictly followed the recommendations of the Guide for the Care and Use of laboratory Animals. Theanimalstudies were approved by the nstitutional Animal Care and Use Committee lACUC) of Xiamen University.The hamster studieswere performed in an animal biosafety level 3 (ABSL-3) laboratory (State Key Laboratory of Emerging nfectiousDiseases,TheUniversitv of Hong Kong).

Note that full information on the approval of the study protocol must also be provided in the manuscript.

Flow Cytometry

Plots

Confirm that:

☒ The axis labels state the marker and fluorochrome used (e.g. CD4-FITC).

☒ The axis scales are clearly visible. Include numbers along axes only for bottom left plot of group (a 'group' is an analysis of identical markers).

☒ All plots are contour plots with outliers or pseudocolor plots.

☒ A numerical value for number of cells or percentage (with statistics) is provided.

Methodology

Sample preparation	Lungs were cut into 0.5-cm pieces, placed in gentleMACS C tubes (Miltenyi) containing collagenase type IV (Gibco) and DNase I (Roche) in PBS containing 2% FBS, and dissociated using a gentleMACS Dissociator (Miltenyi; program m_lung_01). A single cell suspension was obtained by digesting tissue through a 70 μm cell strainer, and centrifugation at 300 g for 5 min at 4 °C. After centrifugation, 1 mL of cold red blood cell lysis buffer (Solarbio) was added for 2 min to lyse red blood cells. The reaction was stopped by adding 10 mL of cold PBS containing 2% FBS and washed once to remove residual red blood cell lysis buffer. Lymphocytes were obtained from the resulting cell suspensions using density gradient centrifugation (Percoll, SIGMA-ALDRICH). Cells were recovered at the interface of the 80% Percoll layer and the 40% Percoll layer, then washed with PBS + 2% BSA at 500 g for 5 min to remove excess Percoll.Cervical lymph nodes were carefully pinched with tweezers and rinsed several times with cold PBS containing 2% FBS. Lymph nodes were ground and passed through a 70 μm cell strainer. Lymphocytes were washed once and resuspended in PBS containing 2% FBS.Mice were euthanized and their spleen were carefully separated and rinsed several times with cold PBS containing 2% FBS. Spleen were ground and passed through a 70 μm cell strainer and the cells were centrifuged at 300 g for 5 min at 4 °C. 10 ml of cold red blood cell lysis buffer (Solarbio) was added, and the samples were incubated for 5 min at 4 °C. The reaction was stopped by adding 20 ml of cold PBS containing 2% FBS and washed once to remove the residual buffer. Lymphocytes were washed once and resuspended in PBS containing 2% FBS.Mouse peripheral blood was transferred into a centrifuge tube containing sodium heparin, then 4 mL PBS buffer was added and transferred to SepMate™ PBMC isolation tubes (STEMCELL). PBMCs used density gradient centrifugation at 1200 g for 10 min at 25 °C (Ficoll-Paque PREMIUM, GE). PBMCs obtained from the middle layer cells.The lower jaw of the mouse was removed, and a surgical knife was used to carefully cut and excise the upper palate by following the inner contour of mouse incisors and molar teeth. The tissue was digested at 37℃ with collagenase type IV (Gibco) and DNase I (Roche) in PBS containing 2% FBS. A single-cell suspension was obtained from the digested tissue using a 70 μm cell strainer.
Instrument	Flow cytometry data with BD LSRFORTESSA X-20 (BD Biosciences) flow cytometry system
Software	Flow cytometry data were analyzed using FlowJo X 10.0.7r2
Cell population abundance	The abundance ofthe post-sort fractions were higher than 98%
Gating strategy	Doublets were excluded using forward light-scatter gating followed by gating on lymphocytes based on FSC/SSC. Deadcellswere excluded by Aqua. These cells were further gated as indicated in Supplementary Figures.

☒ Tick this box to confirm that a figure exemplifying the gating strategy is provided in the Supplementary Information.
